# A Fast and Flexible Framework for Network-Assisted Genomic Association

**DOI:** 10.1016/j.isci.2019.05.025

**Published:** 2019-05-24

**Authors:** Daniel E. Carlin, Samson H. Fong, Yue Qin, Tongqiu Jia, Justin K. Huang, Bokan Bao, Chao Zhang, Trey Ideker

**Affiliations:** 1 Department of Medicine, University of California San Diego, La Jolla, CA 92093, USA; 2 Department of Bioengineering, University of California San Diego, La Jolla, CA 92093, USA; 3 Bioinformatics and Systems Biology Program, University of California San Diego, La Jolla, CA 92093, USA

**Keywords:** Biological Sciences, Genomics, Bioinformatics

## Abstract

We present an accessible, fast, and customizable network propagation system for pathway boosting and interpretation of genome-wide association studies. This system—NAGA (Network Assisted Genomic Association)—taps the NDEx biological network resource to gain access to thousands of protein networks and select those most relevant and performative for a specific association study. The method works efficiently, completing genome-wide analysis in under 5 minutes on a modern laptop computer. We show that NAGA recovers many known disease genes from analysis of schizophrenia genetic data, and it substantially boosts associations with previously unappreciated genes such as amyloid beta precursor. On this and seven other gene-disease association tasks, NAGA outperforms conventional approaches in recovery of known disease genes and replicability of results. Protein interactions associated with disease are visualized and annotated in Cytoscape, which, in addition to standard programmatic interfaces, allows for downstream analysis.

## Highlights

• NAGA is a post-GWAS approach integrating molecular network context
• NAGA maps SNPs to nearby genes and finds network regions that are enriched
• Our approach is fast and flexible, utilizing NDEx for biological interaction networks
• NAGA outperforms a non-network and published GWAS approaches in recovering gene sets

## Graphical Abstract

**Figure:**
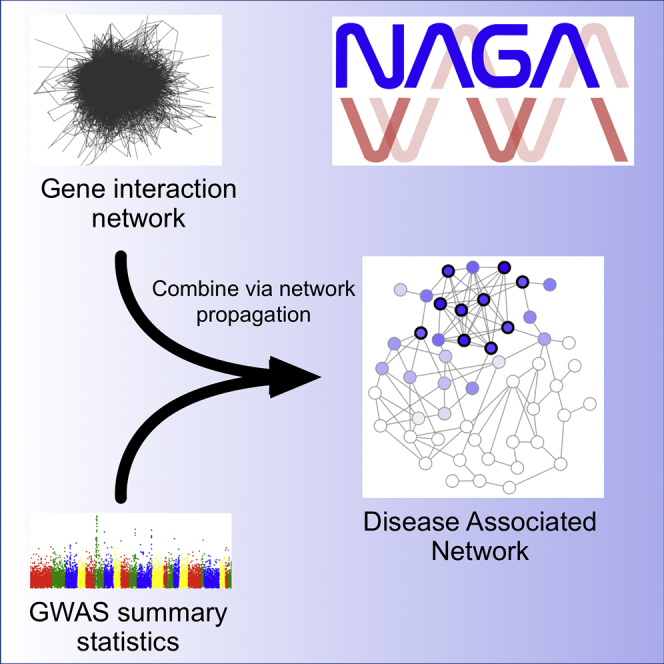

## Introduction

While genome-wide association studies (GWAS) have linked many genetic variants to complex diseases, the variants mapped thus far account for only a small fraction of the total genetic variation affecting any given disease phenotype (Sullivan et al., 2018). A common challenge with these studies is that they typically test millions of single nucleotide polymorphisms (SNPs) for disease association, making it difficult to distinguish the causal loci from the background statistical noise of other variants. This situation leads to the use of very stringent significance thresholds to identify associated variants (e.g., p value < 5 × 10^−8^), with the consequence that all but the strongest findings may be missed (Lander and Kruglyak, 1995).

One recent approach to address this challenge has been to extend the independent analysis of individual variants to more complex models (Visscher et al., 2017), such as polygenic risk scores (PRS), which combine multiple variants in a linear model to predict phenotype (International Schizophrenia Consortium et al., 2009, Wray et al., 2014). However, even these more expansive views do not account for the many non-linear interactions among variants, and these approaches do not attempt to explain how the variants that contribute to the PRS are related to disease mechanisms.

Integration of GWAS studies with protein-protein interactions (PPIs) and other types of molecular networks has recently gained attention as an approach to help overcome the lack of statistical power in the detection of gene-disease associations (Jia and Zhao, 2014). In this regard, many previous approaches have been described for using networks to support GWAS results. An early method was dense-module GWAS (Jia et al., 2011), which scores each protein in a PPI network according to the significance of SNP associations near its encoding gene. Densely connected subnetworks are then identified that locally maximize the proportion of significantly associated proteins. Genome-Wide Association Boosting (GWAB) (Greene et al., 2015, Lee et al., 2011) first construct tissue-specific networks from expression and interaction data, where interactions are weighted based on a tissue-specific Bayesian method. These weights are then used as features of an Support Vector Machine classifier for which the positive class is defined as those genes having genome-wide significant association to a disease. Network-wide Association Studies (NetWAS) (Shim et al., 2017) aims to detect disease-associated genes that have less than genome-wide significance scores according to their proximity to other significant genes in the network using a naive Bayes guilt-by-association approach. Conflux (Mezlini and Goldenberg, 2017) integrates network information as part of a probabilistic graphical model, intended to mitigate noise in the network structure, and then uses a Bayesian framework that allows for setting of disease association probability scores for all genes instead of identifying a fixed set of disease-associated genes. Notably, Conflux uses the variants of individual patients directly, rather than the cohort summary statistics (i.e., p values of association) used by most other approaches.

While these network approaches to GWAS have important differences from an algorithmic standpoint, previous work has shown that the input gene networks also have an important influence on performance (Huang et al., 2018). Unfortunately, many of the previous approaches are dependent on a particular network that is hard-coded, confounding attempts to perform a head-to-head comparison isolating the network GWAS algorithms. Moreover, as new and better molecular network resources are becoming available all the time, one key aspect of any future network GWAS pipeline is its generality with respect to the choice of network. In this respect, the Network Data Exchange (NDEx) database (Pratt et al., 2015) has recently been established for dissemination and exchange of biological networks on the cloud, creating a useful repository of networks for GWAS applications.

Given this state of the field, we set out to address two key requirements that we saw as necessary to facilitate widespread access to network methods by the GWAS community. First, there was a need for an unbiased evaluation framework to identify the best algorithms for network-based GWAS. This first need is, at least in part, addressed by a companion article to this one (Fong et al., 2019). Second, we considered that a simple, lightweight, and performative implementation of network GWAS, compatible with up- and downstream steps in the canonical GWAS pipeline and with easily swappable network choices, should be made available.

Here, we describe an attempt to meet this second need with a software package called Network Assisted Genomic Association (NAGA). NAGA is based on the method of network propagation, which has emerged as a robust and widely used network analysis technique in many bioinformatics applications (Cowen et al., 2017). Insofar as disease variants converge on common sets of interacting genes in a molecular network (also known as pathways), application of network propagation to GWAS distributes the effects of variants at each genomic locus to network neighbors. For variants affecting the same network region, the result is variant aggregation and amplification of signal.

## Results

### Overview

**Figure 1:**
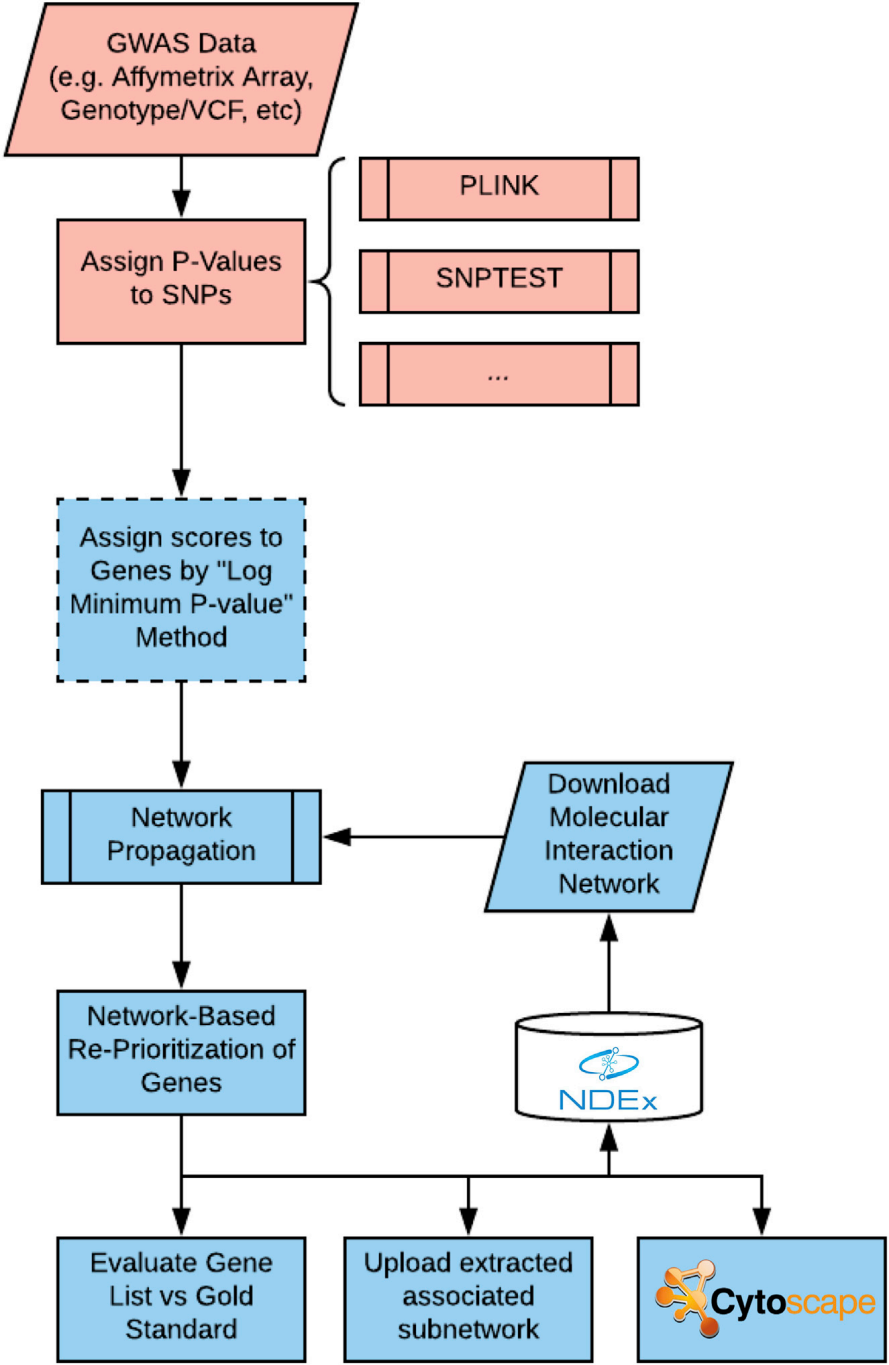
NAGA Workflow Red steps are upstream of the method; blue steps are provided by the NAGA python package.

The NAGA approach involves a straightforward multistep procedure (Figure 1). Our method starts with summary p values assigned by PLINK (Chang et al., 2015), SNPTEST (Marchini et al., 2007), or another standard GWAS analysis approach. The first step is to assign each gene a score corresponding to the p value of the most significantly associated SNP within a genomic window. Second, a molecular network is then downloaded from the NDEx database and integrated with these gene scores. Third, the technique of network propagation is performed to spread the gene scores to network neighbors, resulting in revised scores that are used to prioritize all genes in a final ranked list. Finally, this ranked gene list may be validated and explored using a variety of means, including comparison to a gold-standard set of genes to establish that NAGA has enriched for biological processes of interest. Another endpoint is to create subnetwork(s) implicated by the prioritized variants, which can then be published to NDEx for sharing and publication. The full NAGA pipeline is available as a Jupyter notebook and is also available via REST API. Source code and information on API access can be found at https://github.com/shfong/naga. Details of each step of the procedure are in Transparent Methods.

### Evaluation

**Figure 2:**
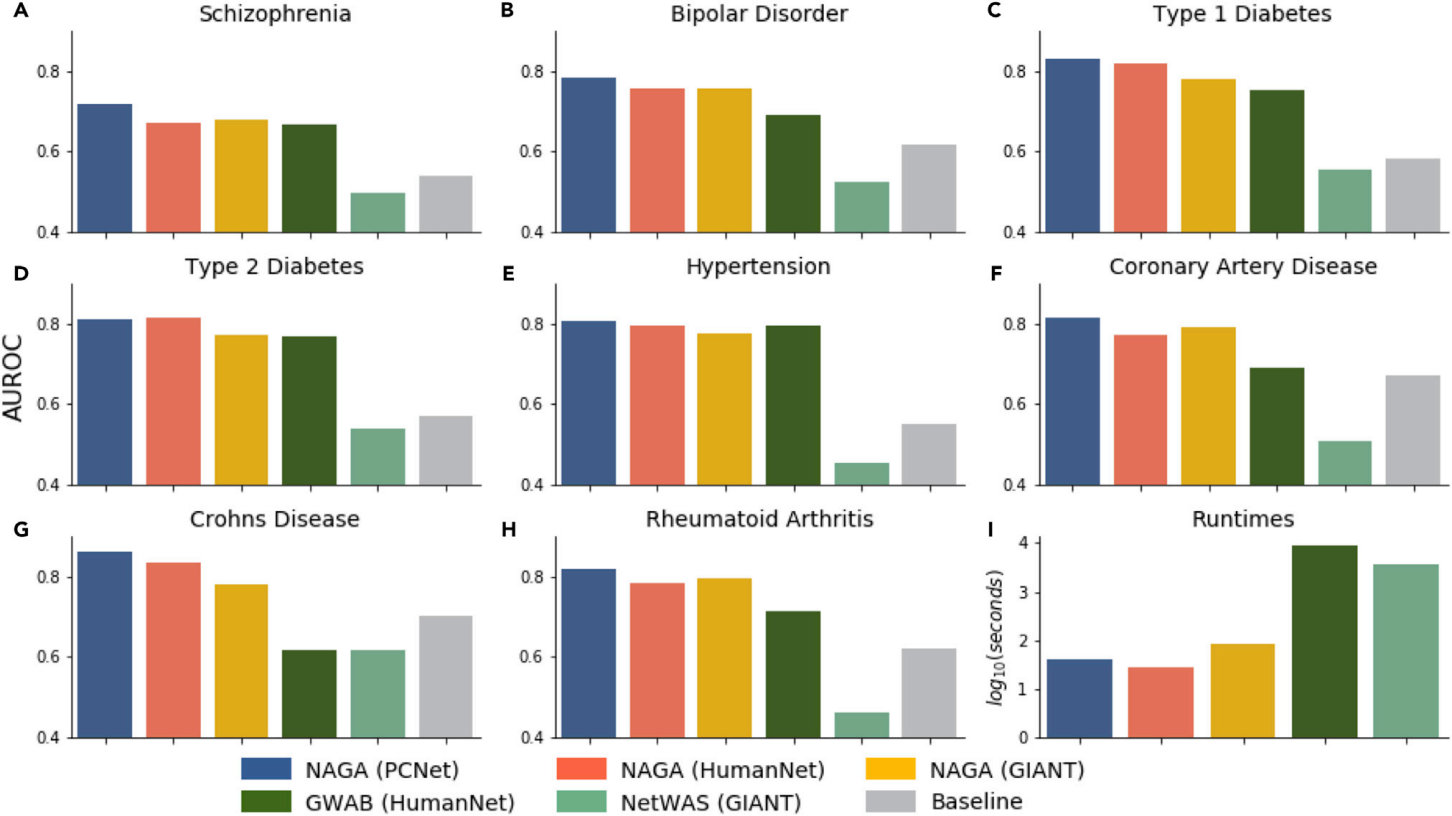
AUROC Results against Gold-Standard Disease Genes Area under the receiver-operator curve (AUROC) for three different network GWAS methods, using the gene network shown in parentheses for (A) Schizophrenia, (B) Bipolar Disorder, (C) Type 1 Diabetes, (D) Type 2 Diabetes, (E) Hypertension, (F) Coronary Artery Disease, (G) Crohn's Disease, and (H) Rheumatoid Arthritis. (I) Runtime for the methods.

We evaluated the performance of NAGA and two other network-based methods, NetWAS (Greene et al., 2015) and GWAB (Shim et al., 2017), in analysis of a schizophrenia GWAS dataset (Schizophrenia Psychiatric Genome-Wide Association Study (GWAS) Consortium, 2011) with 9,394 cases and 12,462 controls. This original study found seven loci that reached global significance. Performance was evaluated using a hypergeometric test of the top 100 genes returned by each method against a literature meta-analysis schizophrenia gene set, made up of 1,147 genes published before publication of the GWAS (Allen et al., 2008). The hypergeometric test evaluates whether the overlap between the top 100 returned genes and the literature gene set is significant. The performance scores and runtimes of NAGA using three different human genome-scale networks—PCNet, HumanNet v2 (Hwang et al., 2019) (used by the GWAB method), and GIANT (Shim et al., 2017) (used by the NetWAS method, non-tissue specific)—were compared with GWAB and NetWAS (Figure 2). NAGA applied to all networks significantly enriched for the schizophrenia gold-standard set of genes. NAGA using PCNet performed best of all approaches, recovering 33 gold-standard genes in its top 100 (hypergeometric p value < 10^−27^). Expressed as an area under the receiver-operator curve (AUROC), NAGA achieved an AUROC of 0.72 (Figure 2A). The baseline method, where we simply mapped p values to genes and ranked genes according to their maximally significant variant, did not find a significant enrichment over background among its top 100 calls and achieved an AUROC of 0.54. The top GIANT network and the NetWAS method also significantly enriched for literature-curated genes among their top 100.

To investigate whether the above results were specific to a single schizophrenia GWAS or whether they were applicable to GWAS in general, we repeated the above workflow with seven additional GWAS made available by the Wellcome Trust Case Control Consortium (2007) (Figures 2B–2H) for bipolar disorder, type 1 diabetes, type 2 diabetes, hypertension, coronary artery disease, Crohn disease, and rheumatoid arthritis. For the reference gene sets, we used the corresponding gene sets from DisGeneNET (Piñero et al., 2017), which integrates expert-curated and text-mined disease associations. We found that in all eight GWAS (including the schizophrenia study above), NAGA yielded the best results out of the three network approaches by AUROC. In addition, in seven of the eight, the default setup using PCNet was the best performer, and in the other case (type 2 diabetes) NAGA using the HumanNet won out.

We found that the NAGA method runs relatively quickly, likely related to its algorithmic simplicity. Configured with PCNet and used to analyze the schizophrenia cohort, NAGA completed in less than 5 minutes on a mid-2015 Macbook Pro with 16-GB RAM (Figure 2I). This performance was very favorable when compared with those of NetWAS and GWAB, which required 1 and 2.4 h, respectively (Figure 2I). As one caveat of this analysis, code was not publicly available for the other tools, thus their runtimes were based on web-accessible versions. For this reason, runtime estimates may contain significant computational overhead such as waiting in queues and data transfer to and from the servers. In addition, these methods build a model that must be evaluated for each gene separately, whereas the NAGA calculation can be performed for all genes simultaneously. Thus NAGA can complete a network GWAS analysis in a few minutes on a modern laptop.

**Figure 3:**
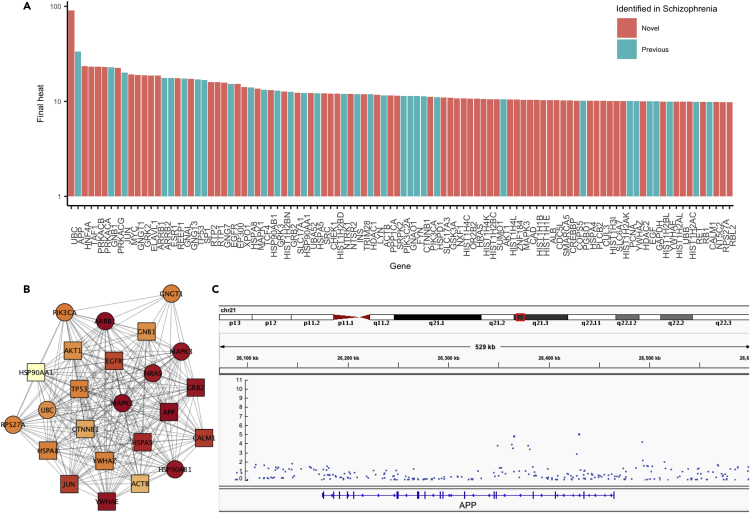
Application of NAGA to Schizophrenia (A) Top 100 prioritized genes after network propagation of a schizophrenia GWAS dataset. Genes in the gold standard are represented by turquoise bars, whereas newly implicated genes are represented by red bars. (B) Subnetwork associated with hottest network propagation scores. Subnetwork is visualized with the initial association scores mapped to node colors, with darker red corresponding to stronger association. Previously implicated schizophrenia genes appear as squares, and newly implicated genes appear as circles. (C) Integrated Genomics Viewer (IGV) screenshot showing the genomic locus of APP, the second highest scoring gene from (A). IGV displays the log 10 p-value of association. APP contains SNPs that, before network propagation, achieve nominal but not global statistical significance of association.

To closely examine an example gene association boosted substantially by network analysis, we delved deeper into the schizophrenia result, looking at the top 100 genes returned by the pipeline (Figure 3A). We visualized the APP gene locus, which was the second-ranked gene in our analysis, using Integrated Genomics Viewer (Robinson et al., 2011) (Figure 3B). Although SNPs at the APP locus were nominally significant (min p value within 10 kb = 9.56×10^−6^), none made the genome-wide significance cutoff. We examined regions of PCNet impacted by the top 100 results using the ModuLand network clustering App in Cytoscape (Szalay-Beko et al., 2012) (Figure 3C). One of the network clusters contained APP along with several previously implicated schizophrenia genes. Owing to the nominal association of several APP network neighbors (MAPK1, ARRB1, YWHAE, HSP90AB1), APP itself was implicated despite not reaching genome-wide statistical association originally. Notably, APP has been implicated in a number of neural disorders, including Alzheimer disease and intellectual disability (Myrum et al., 2017).

## Discussion

We have demonstrated a fast and flexible solution for network-based GWAS. The direct connection with the NDEx and Cytoscape platforms allows new molecular networks to be used in the pipeline as soon as they are published to the resource, lowering the barrier to translating new network results into genome interpretation.

Although other network query services such as GeneMANIA (Mostafavi et al., 2008) and STRING (Szklarczyk et al., 2016) have existed for some time, our system is especially suited for GWAS analysis. Specifically, we address the question of SNP-to-gene mapping and scoring in addition to network propagation and allow for different genomes and networks. Although GeneMANIA also provides for custom network uploads, it neither provides for continuous value query scores such as the log-10 transform we use in this work nor returns continuous output values for the whole genome allowing for the area under the curve calculation that was used for evaluation here. In our companion article (Fong et al., 2019), we show that the use of continuous scores is advantageous in the schizophrenia example. Also in the companion article, we evaluated several different approaches to network-boosted GWAS, including different scoring schemes, propagation algorithms (including heat diffusion), and network settings.

### Limitations of Study

Given its conceptual and mathematical simplicity, the success of network propagation in the setting of network GWAS is striking and provides a point of departure for further bioinformatics methods development in this area. Conflux, which uses a more complex Bayesian graphical model, shows positive results when compared with network propagation on simulated networks, and on small real networks with simulated data (Mezlini and Goldenberg, 2017). However, in addition to hard-coding a preferred network, Conflux currently only operates on small networks because of the computational overhead of the Bayesian model. Conflux has another feature that both adds power, on the one hand, and limits its broad application on the other; it uses patient-level variant data rather than summary statistics for its calculations (such as the log p value used here, or the effect size of chi-square tests of association). This feature is clearly advantageous, as it allows statistical interactions to contribute to the association of sets of genes to a phenotype; more efficient methods along these lines will be welcome in future studies.

Along these lines, we see room for many creative approaches in network analysis of variants at the patient level. For instance, one might first apply network propagation on the whole gene network to implicate smaller subnetworks and then use a patient-level method like Conflux to train the final model on that smaller subnetwork. This approach would rely on flexibility in the choice of networks, because each new cohort would generate a new implicated subnetwork.

It should be noted that the procedure for mapping association scores to genes is an important factor in network GWAS techniques that we have not extensively explored here. For instance, PEGASUS finds an analytical model for the expected chi-square statistics because of correlation from linkage disequilibrium, which worked well with the network propagation algorithm HotNet2 (Leiserson et al., 2015, Nakka et al., 2016). Transcriptome-wide association studies (Gusev et al., 2016) explicitly model expression quantitative trait loci and derives an association score between the gene's inferred expression and the phenotype. The results of these other mapping methods can also be used instead of the simple method based on gene distance explored here.

## Methods

All methods can be found in the accompanying Transparent Methods supplemental file.

## Data and Software Availability

Source code and information on API access can be found at https://github.com/shfong/naga.

NAGA can be run from a web service, found at http://nbgwas.ucsd.edu/.

The networks used in this paper can be found on the NDEx database:

PCnet: http://www.ndexbio.org/#/network/f93f402c-86d4-11e7-a10d-0ac135e8bacf.

GIANT: http://www.ndexbio.org/#/network/08ba2a31-86da-11e7-a10d-0ac135e8bacf.

HmanNet: http://www.ndexbio.org/#/network/18dc9109-86da-11e7-a10d-0ac135e8bacf.

## Supplemental Information

Document S1. Transparent Methods and Figure S1
