# Supplementary material for: A Fast and Flexible Framework for Network-Assisted Genomic Association
Source: iScience. 2019 May 24;16:155–61. doi: 10.1016/j.isci.2019.05.025 (PMC6554232; doi:10.1016/j.isci.2019.05.025)
Supplement: Document S1. Transparent Methods and Figure S1 [file mmc1.pdf]

ISCI, Volume *16*

Supplemental Information

A Fast and Flexible Framework for Network-Assisted Genomic Association

Daniel E. Carlin, Samson H. Fong, Yue Qin, Tongqiu Jia, Justin K. Huang, Bokan Bao, Chao Zhang, and Trey Ideker

Transparent Methods

Key Resources Table

REAGENT or RESOURCE	SOURCE	IDENTIFIER
**Deposited Data**		
Psychiatric Genomics Consortium (PGC)	Schizophrenia Psychiatric Genome-Wide Association Study (GWAS) Consortium (2011)	https://www.med.unc.edu/pgc/results-and-downloads
WTCC1	Wellcome Trust Case Control Consortium (2007)	https://www.wtccc.org.uk/
**Software and Algorithms**		
NAGA	This paper	https://github.com/shfong/naga
Cytoscape	Shannon et al. 2003	https://cytoscape.org/
IGV	Robinson et al. 2011	http://software.broadinstitute.org/software/igv/

Method Details

Assigning gene association scores. The approach begins with GWAS summary statistics (e.g. chi-squared p-values of association with the phenotype) on SNPs or other types of variants in the genome. We then define regions of SNPs for assignment of p-values to coding genes. Specifically, for each gene we define a region including the gene body and a specified number of kilobases up- and downstream of the gene and assign the smallest p-value in that region. Herein we use a window of ±10 kb, although the window size is customizable. We then take the largest −log(p-value) assigned to the gene as the gene score. Choosing the minimum p-value within a 10 kb window is the same mapping strategy employed by GWAB (Lee et al., 2011).

We found that overall performance of NAGA was robust to window size (**Supplementary Figure S1**); this conclusion was based on experiments conducted using the Wellcome Trust GWAS data (Wellcome Trust Case Control Consortium, 2007) to recover gold standard gene sets cataloged by the DisGeNET project (Piñero et al., 2017). In these experiments, we looked for enrichment of disease-associated genes from DisGeNET among the genes with significant p $<10^{-6}$. On the Wellcome Alzheimer's data, we also compared genes ranked by network GWAS to a differentially expressed gene set for the same disease (Castillo et al., 2017). While different datasets yielded different best window sizes, we found that 10 kb was a reasonable default choice resulting in near-optimal precision and recall for the majority of diseases.

Network Selection. For the default network, we chose PCNet since this network has been shown to perform well at diverse network propagation tasks (Huang et al., 2018). PCNet

contains 19,781 genes connected by approximately 2.7 million edges. However, any network available in the NDEx database is easily accessible by specifying the UUID of the network on the public server (http://www.ndexbio.org). These UUIDs are available by going to the NDEx public server and searching for the desired network. Alternatively, users can upload their own networks to NDEX, in which case the UUID is assigned on upload. Nearly all public networks, including STRING (Szklarczyk et al., 2016) and GIANT (Greene et al., 2015), are available in NDEx. Instead of pinning the analysis to a single network, or a series of networks formed by the same method, users have easy access to thousands of diverse networks for analysis. Similarly, the users have the option to upload results as annotated networks to NDEx and share the results with collaborators.

Network Propagation. Gene scores were propagated across a molecular network to diffuse the effect of these mutations to the surrounding network neighborhood. Genes that are near query nodes are implicated by association. For a review of the many flavors and applications of network propagation, see (Cowen et al. 2017). Of the several variations, here we use the random walk with restart model (Vanunu et al. 2010). This variant has been shown to work well in analyzing GWAS and cancer variants in the past (Hofree et al., 2013; Huang et al., 2018). Its central equation is:

$$F(t+1) = (1-\alpha) * F(t)A + \alpha * F(0)$$

This model accepts a propagation constant (α), the gene mutation profiles for a phenotype ($F(0)$), and a degree-normalized adjacency matrix representing the network (A). Thus at every time step, there is some (equal) probability of walking to the network neighbors, and also some probability (given by α) of resetting to the original gene score profile described above. When propagated to convergence as $t \rightarrow \infty$, this model yields a propagated profile of genes (F) summarizing the overall effect of gene mutations across the network. α is set by a linear model determined by network density (Huang et al., 2018). By reranking genes according to this final heat, we obtain a new reprioritized list of genes based on significant associations present in the network neighborhoods.

Visualization and further analysis of subnetwork results. One of the goals of NAGA is to present a general and flexible pipeline, so that users can leverage existing network resources and utilities. In addition to sourcing networks from NDEx (Pratt et al. 2015) as described above, NAGA leverages Cytoscape (Shannon et al. 2003) for exploring network results (Figure 1). We have hooked the NAGA pipeline into Cytoscape using CyRest (Ono et al. 2015), allowing users to interact with the molecular subnetworks that underpin the results. In addition to the interactivity of Cytoscape, users can also invoke hundreds of popular apps in Cytoscape to annotate, visualize, cluster and interpret the network.

Variations. We have found a second gene score transform that also performs well in different contexts; this second approach simply binarizes the significant gene hits according to an adjustable cutoff. For this setting we use a default setting of 5×10^{-6}. Genes that are more significant than this cutoff are “query genes” assigned an initial value, which defaults to 1, while all other genes in the genome are assigned a 0. We have also implemented heat diffusion as a second algorithmic option. Heat diffusion is similar to random walk, but instead of having a probability of reseting and running to steady state, as is the case in random walk with restart, heat diffusion performs the random walk for a certain amount of time without restart. The user can define the time interval, which defaults to 0.1 based on previous work, to diffuse query genes (Carlin et al. 2017).

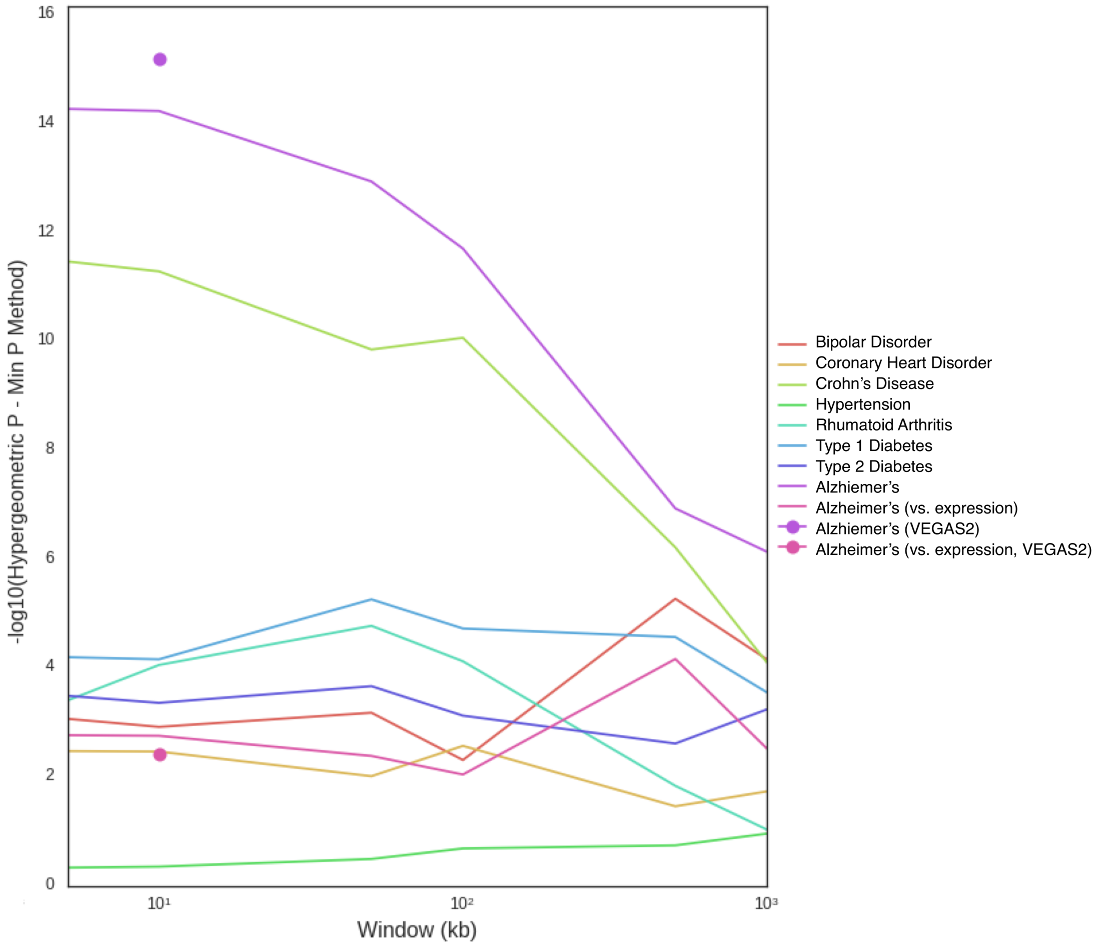

Supplemental Figure 1, Related to Figure 2. Enrichment for DisGenNET gene sets in Wellcome GWAS associations for different choices of genomic window size around genes.
